# Sigmoid Sinus Thrombosis As Complication of Otitis Media in a 3-Year-Old Boy: Case Report and Review of the Literature

**DOI:** 10.7759/cureus.22262

**Published:** 2022-02-15

**Authors:** Nektaria Kalyva, Vasileios K Mousafeiris, Aristeidis Giannakopoulos

**Affiliations:** 1 Pediatrics, University Hospital of Patras, Patras, GRC; 2 Pediatrics, Brookdale University Hospital Medical Center, New York, USA

**Keywords:** sigmoid sinus thrombosis, mastoiditis, antibiotics therapy, myringotomy, recurrent infection, cerebral venous sinus thrombosis (cvst), cerebral venous sinus, acute otitis media

## Abstract

Sigmoid sinus thrombosis is a relatively rare, but severe complication of acute otitis media and mastoiditis among other conditions. We report a case of a 3-year-old boy with a history of recurrent acute otitis media which was initially partially treated with antibiotics for 1 month before his admission to our department for high fever and headache. Although initially, no signs of central nervous system (CNS) involvement were present, clinical suspicion for CNS pathology led our whole work-up to conclude the diagnosis of sigmoid sinus thrombosis. The patient was subsequently treated with intravenous antibiotics, anticoagulation therapy and also underwent myringotomy, bilateral tympanostomy tube placement, and mastoidectomy. Cerebral sinus thrombosis is a life-threatening condition that usually complicates the neglected acute otitis media or mastoiditis. Optimal treatment includes antibiotic therapy, hydration, and pain management, with the debatable role of anticoagulation therapy and mastoidectomy.

## Introduction

Sigmoid sinus thrombosis (SST) is a potentially life-threatening, although rare, clinical condition, that can affect both neonates and children [1]. In neonates, it is usually preceded by acute systemic illness such as sepsis or respiratory failure, whereas in children, it may follow acute head or neck infections such as acute otitis media (AOM) [2].

Herein, we report the case of a 3-year-old boy with unilateral sigmoid sinus thrombosis (SST) as a complication of recurrent ipsilateral otitis media. Recovery was not uneventful as soon thereafter, the patient developed intracranial hypertension which needed the placement of a ventriculoperitoneal shunt. We present this case because of the rarity of complex sequelae and we discuss the etiology, predisposing factors, and natural evolution of SST due to AOM. 

## Case presentation

A 3-year-old boy was admitted to our Emergency Room (ER) with 3 days of fever up to 39.7^ ο^C and otalgia. One month prior to his admission, he was diagnosed with recurrent AOM and was treated initially with oral amoxicillin 80mg/kg/day for 10 days by his primary care pediatrician. After a short remission, symptoms re-emerged, and he was treated with a combination of oral amoxicillin/clavulanic acid 45mg/kg/day and amoxicillin 45mg/kg/day for 4 days, which was stopped due to a reported allergy and continued on clarithromycin 15mg/kg/day for another 5 days. The patient was complaining of a headache that was not improving with either acetaminophen or ibuprofen and was accompanied by a few episodes of non-bloody, non-bilious vomit for four days before his ER visit. His mother also noticed drowsiness the day preceding his ER visit.

On physical examination, the patient appeared tired, inactive, and pale, but not in distress. His vital signs were within normal limits (Heart rate: 102/min, Blood pressure: 93/48mmHg, Temperature: 36.2 ^ο^C, O_2_ sat: 99%). The tympanic membranes bilaterally were erythematous and bulging. His oral cavity was erythematous and small cervical lymph nodes were palpated bilaterally. His score on the Glasgow Coma Scale (GCS) was 15/15 and there were no signs of meningeal irritation.

The initial workup included laboratory tests (complete blood count, comprehensive metabolic panel, CRP, blood culture) which showed a WBC of 21910/mm^3^ and a CRP value of 30.9 mg/dl (normal range: <0.5). Comprehensive metabolic panel values were within the normal range. The patient was also evaluated by a pediatric ENT physician who clinically excluded the presence of mastoiditis bilaterally. Imaging tests such as CT or MRI of the head were not suggested and not performed at this point. The patient was then admitted to our pediatric department. Intravenous (IV) antibiotic treatment with ceftriaxone (100mg/kg/day) was administered because of failed outpatient oral antibiotic treatment twice and the patient’s reported allergy to amoxicillin/clavulanic acid.

Our patient, initially, responded well to the IV antibiotic treatment; he remained afebrile for the first 2 days and was clinically improving. Unexpectedly, on the third day of admission, the patient’s clinical status deteriorated. He became irritable with alternating drowsiness and severe headache. Fundoscopy was performed that showed no fundus or optic nerve edema or hemorrhage. An emergent head CT without contrast was performed that excluded an intracranial hemorrhage or cerebral infection. However, it showed the increased size of the right sigmoid sinus in comparison to the left. CSF analysis was normal (appearance: clear, colorless, cells 12, RBCS 1, PMNs 4, Lymphocytes 5, Monocytes 3, Gram stain: negative, Ziehl-Neelsen stain: negative for acid-resistant and alkali-resistant bacteria, glucose 60 mg/dl, total proteins 20.8 mg/dl., CSF culture: negative). The pediatric ENTs were again consulted, and bilateral myringotomy was performed with insertion of tympanostomy tube bilaterally; culture of ear fluid was also sent. At this time point (day 4 of admission) antibiotic coverage was upgraded by substituting ceftriaxone with IV meropenem (120mg/kg/day) and vancomycin (60mg/kg/day). In the next 24 hours, both clinical (resolution of symptoms) and laboratory improvement (reduced WBC: 9540/ mm3 and reduced CRP: 11.67 mg/dl) were noticed. 

**Figure 1:**
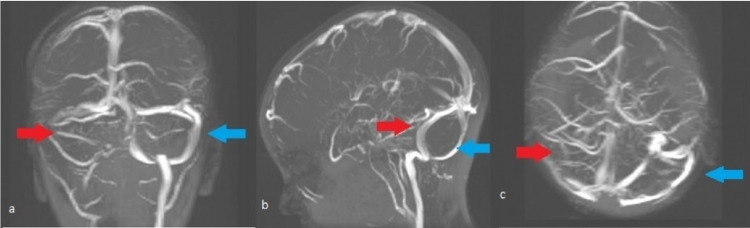
MRI-MRV cross-sections (a. coronal, b. sagittal, c. transverse) After the administration of a paramagnetic substance, a filling defect is noted in the final part of the right internal jugular vein and the sigmoid sinus on the same side. (Red arrow depicts the filling defect, Blue arrow depicts the normal contralateral side)

Subsequent imaging with MRI-MRV of the brain showed findings consistent with SST (Figure 1).

Furthermore, fluid was recognized in the mastoid cells bilaterally, a finding consistent with possible mastoiditis. The brain parenchyma, ventricles, subarachnoid space, and meninges were normal. After confirmation of the diagnosis, the patient was transferred for pediatric neurosurgery and ENT management. Subsequently, the patient underwent simultaneous bilateral mastoidectomy (day 8 of admission) and was also put on anticoagulation treatment for six months with simultaneous 6-week antibiotic therapy. However, while on anticoagulation therapy, on day 14 of admission, he developed signs of increased intracranial pressure and hydrocephalus which required the placement of a ventriculoperitoneal shunt. Thereafter, the patient showed signs of clinical and laboratory improvement and -resolution of his symptoms and was subsequently discharged. Follow up visits at 3 and 6 months revealed complete recovery and an MRI performed at 6 months showed complete resolution of his sigmoid sinus thrombosis (SST).

## Discussion

SST is a severe cerebrovascular condition affecting children in Europe and North America with an estimated annual incidence of 0.6 per 100,000 [1]. Thrombosis of cerebral veins or sinuses obstructs the blood drainage from the brain tissue, leading to decreased cerebrospinal fluid (CSF) absorption, elevated intracranial pressure, and cerebral parenchymal lesions [2].

SST is most commonly seen in neonates and children [3,4]. In neonates, acute systemic conditions such as hypoxia, ischemia, respiratory depression, sepsis, gastrointestinal pathology, or dehydration have been the most common causes of SST [1,5]. In previously healthy children, however, head and neck infections such as acute otitis media, mastoiditis, and sinusitis have been shown to be the most common underlying conditions that lead to the development of SST [1,3,5]. Other less common causes such as anemia, hemoglobinopathies, prothrombotic states, lupus, diabetes, nephrotic syndrome, inflammatory bowel disease, and cancer have also been reported. Ichord et al. found a prothrombotic condition in 20% of patients with SST [3].

The diagnosis of SST requires a high index of clinical suspicion as the signs and symptoms can be variable and, in many cases, are vague or non-specific. Children usually present with a constellation of symptoms like fever, otalgia, headache, vomiting, and depressed mental status as in our case [6-8]. As the condition progresses, increased intracranial pressure (ICP) can present as cranial nerve palsies, papilledema, altered mental status, and can even lead to seizures, stupor, and coma [6-8].

In previously healthy children with acute otitis media as the predisposing factor for SST, the presence of mastoiditis is crucial in optimizing the therapeutic approach. Several studies found that acute otitis media and mastoiditis were present in 37.5-47% of cases with SST [3,7]. Thus, we should always assess for mastoiditis in patients with SST as a complication of AOM.

Timely diagnosis and treatment are critical for the outcome of SST. Imaging modalities of choice are CT angiography or MRV, as normal head CT cannot exclude SST [6,7].

Management in the acute phase of SST consists of pain management, IV antibiotics, hydration, and initial aggressive anticoagulation therapy [8]. A high clinical index of suspicion for lack of response to initial treatment must be maintained, as in our case, where the patient deteriorated after the initial treatment. Measures to control ICP such as acetazolamide and lumbar puncture may be necessary if there is no response to optimal treatment. Anticoagulation therapy may be continued after the acute phase for a period of time that is dictated by the presence of other comorbidities [9].

Anticoagulation treatment has been under debate in the recent literature. Few studies report better neurologic outcomes for those patients treated with anticoagulation [10,11]. On the other hand, there are studies that show no improvement with the use of anticoagulation or even report major side effects of anticoagulation use in the treatment of SST [12,13].

Furthermore, surgical intervention can play a role in the management of such cases. Μastoidectomy has been the gold standard for the treatment of cholesteatoma and chronic suppurative otitis media [9,14]. Singh et al. reported two cases of chronic suppurative otitis media (CSOM) with exacerbation of AOM, complicated by mastoiditis and septic lateral sinus thrombosis, similar to our case. They managed the patients with mastoidectomy and antibiotic treatment and they reported excellent outcomes [9]. However, to the best of our knowledge, it is still debatable as to which therapeutic approach to follow when there is an intracranial complication of the AOM or CSOM.

## Conclusions

To conclude, we cited antibiotic treatment failure as a cause for retracted suppurative otitis media that led to mastoiditis and SST. It is true that our patient also had active ear discharge, adding the ASOM as a further risk factor for mastoiditis as previously reported. Existing controversy on the implementation of anticoagulation therapy, as well as the choice of surgical intervention for the management of SST further complicates their outcome. We hope that this case report will bring up this rare nosology (SST) as a consequence of a very common pediatric problem (AOM) and add to the clinical experience.
